# Family physicians' effort to stay in charge of the medical treatment when patients have home care by district nurses. A grounded theory study

**DOI:** 10.1186/1471-2296-10-45

**Published:** 2009-06-22

**Authors:** Sonja Modin, Lena Törnkvist, Anna-Karin Furhoff, Ingrid Hylander

**Affiliations:** 1 Department of Neurobiology, Care Science and Society, Centre for Family and Community Medicine (CeFAM), Karolinska Institutet, Alfred Nobels allé 12, S-14284 Huddinge, Sweden

## Abstract

**Background:**

District nurses (DNs) provide home care for old persons with a mixture of chronic diseases, symptoms and reduced functional ability. Family physicians (FPs) have been criticised for their lack of involvement in this care. The aim of this study was to obtain increased knowledge concerning the FP's experience of providing medical treatment for patients with home care provided by DNs by developing a theoretical model that elucidates how FPs handle the problems they encounter regarding the individual patients and their conditions.

**Methods:**

Semi-structured interviews were conducted with 13 Swedish FPs concerning one of their registered patients with home care by a DN, and the treatment of this patient. Grounded theory methodology (GTM) was used in the analyses.

**Results:**

The core category was the effort to stay in charge of the medical treatment. This involved three types of problems: gaining sufficient insight, making adequate decisions, and maintaining appropriate medical treatment. For three categories of patients, the FPs had problems staying in charge. Patients with reduced functional ability had problems providing information and maintaining treatment. Patients who were "fixed in their ways" did not provide information and did not comply with recommendations, and for patients with complex conditions, making adequate decisions could be problematic. To overcome the problems, four different strategies were used: relying on information from others, supporting close observation and follow-up by others, being constantly ready to change the goal of the treatment, and relying on others to provide treatment.

**Conclusion:**

The patients in this study differed from most other patients seen at the healthcare centre as the consultation with the patient could not provide the usual foundation for decisions concerning medical treatment. Information from and collaboration with the DN and other home care providers was essential for the FP's effort to stay in charge of the medical treatment. The complexity of the situation made it problematic for the FP to make adequate decisions about the goal of the medical treatment. The goal of the treatment had to be constantly evaluated based on information from the DN and other care providers, and thus this information was absolutely crucial.

## Background

In Sweden as well as in most Western countries, the shift from hospital care to community and primary care for the ageing population has increased the need for home care. Thus, home care is of growing importance [1,2]. Patients with home care are typically elderly persons with complex problems including a mixture of chronic diseases and symptoms and reduced functional ability [3-5], and they have a significant need for medical treatment. In contrast to what could be expected, however, we found in a previous study of patients with home care provided by district nurses (DN) that the family physicians (FP) saw these patients less often than other patients of comparable age seen by the FPs [3]. This gives rise to questions concerning how the need for medical treatment is met for patients receiving home care.

Home care as a phenomenon is defined as "the care provided by professionals to people in their own homes with the ultimate goal of not only contributing to their quality of life and functional health status, but also to replace hospital care with care in the home for social reasons; home care covers a wide range of activities, from preventive visits to end-of-life care" [6]. Care provided to patients in their homes by DNs is one type of home care in Sweden. Different organisations and different professional actors are involved in parallel in the care of these patients, and much of the medical care is provided outside the home, either at healthcare centres or in hospital [7]. In a study from the UK, it was reported that FPs could experience older people's multiple pathology as complex and sometimes threatening, and that FPs could consider changes in community care as a problem because their workload increased as a result [8]. We have not found any studies that explore how FPs handle their responsibility for the medical problems of patients with home care provided by DNs, or how the complex problems of patients with home care influence the way in which FPs provide medical treatment.

Home care in Sweden is a responsibility of both the municipalities and the county councils. The municipalities are responsible for home care in the form of subsidized home help service. Home care provided by DNs is a responsibility of the county councils and is part of primary care. However, approximately half of Swedish municipalities have taken over that responsibility to simplify collaboration with home help providers. Thus, the DNs work either at a healthcare centre together with the FPs or in a separate organisation from that of the FPs. DNs have undergone specialised training, but nurses without specialised training and assistant nurses under the supervision of a district nurse also work in home care. In this article "DN" includes all nurses and assistant nurses working in home care.

The FPs (specialised in family medicine) are responsible for the medical treatment in primary care for those patients registered with the FP, including those who receive home care from DNs. The FPs are also expected to coordinate treatment prescribed elsewhere, such as by specialised care or inpatient hospital care. One FP may take over responsibility for these patients from the other FPs at a healthcare centre.

The aim of this study was to obtain increased knowledge concerning the FP's experience of providing medical treatment for patients with home care provided by DNs by developing a theoretical model that elucidates how FPs handle the problems they encounter regarding the individual patients and their conditions.

## Methods

The medical treatment of patients with home care provided by DNs is an important but largely unknown process. We explored this process from the FP's point of view using a qualitative method, grounded theory methodology (GTM), where data were collected through interviews with FPs [9]. The setting was primary care in Sweden, where patient conditions in home care vary substantially. The FPs were asked to talk about a patient, listed with them, who had home care provided by DNs.

Thirteen FPs were interviewed. Among them, seven were men and six were women, their ages ranged from 36 to 58 years, they had worked between one and 20 years as a specialised FP, and between six months and 13 years at the present healthcare centre. Both privately owned and county council owned healthcare centres were included. Some FPs (3) worked in different, but most (10) worked in the same organisation as the DN. FPs were included who worked in the city centre, in nearby and more distant suburban areas, but not in rural areas. The number of FP positions at the healthcare centres varied (4–15), as did the number of FP positions filled by FPs (32–105%). The number of DN positions also varied (2–8) (two FPs did not know this figure), as did the number of positions filled (33–100%). The number of patients with home care by DNs for whom the individual FP was responsible also varied (Table 1). Further, two FPs had taken over responsibility from the other FPs at their respective healthcare centres for all patients with home care provided by DNs. Thus these two healthcare centres had a special home care FP. For these FPs, the medical treatment of patients with home care by DNs was a substantial part of their day to day work. The sample of patients the FPs chose to talk about is described in table 2.

**Table 1:** The interviewed family physicians and the patients registered with them who received home care by district nurses

**Label following quotes**	**Number of family physicians**	**Number of registered patients with home care**	**Special home care family physician**
A	2	50–60	Yes
B	5	20–35	No
C	4	< 10	No
D	2	Did not know	No
	∑ 13		

**Table 2:** The sample of patients included in this study

	**Age**	**Sex**	**Medical, functional and other problems encountered**
1	-	Female	Depression, Pain, Overuse of painkillers
2	61	Male	Alcohol abuse, Epilepsy, Dementia
3	> 75	Female	Dementia, Pain, Epilepsy?
4	78	Female	Depression, Dementia and Aphasia after stroke, Incontinence
5	82	Male	Impaired peripheral circulation, Ulcers, Pain
6	85	Female	Asthma, Diabetes, Dementia, Infections
7	86	Male	Prostate hypertrophy, Uraemia
8	87	Female	Dementia, Heart failure, Incontinence, Diabetes
9	87	Male	Diabetes, Obesity, Neuropathy, Both legs amputated, Ulcers, Infections, Pain
10	87	Male	Metastasized kidney cancer, End of life care
11	89	Female	Glaucoma, Bad eyesight, Aortic stenos, Dizziness and falls, Fractures
12	89	Female	Severe anaemia, Leg ulcers
13	90	Male	Diabetes, Arthrosis, Heart failure, Spanish speaking
14	95	Female	Aged, Deteriorating health, Pneumonia, End of life care
15	Old	Male	Aged, Heart failure, Angina, Prostate hypertrophy, Dizziness and falls

### Theoretical sampling

The sampling procedure was conducted in accordance with GTM [10], i.e. theoretical sampling, which means that the collection of data is done continuously and in interaction with data analyses.

For a first sample, an invitation was issued to three healthcare centres, and one FP from each centre agreed to participate. Before each interview the project was presented in a postal invitation. The FPs in the first interviews (interviews 1–3) were informed that they were expected to talk about the last patient in whose medical treatment they had been involved who had home care provided by a DN. The patient was to be 65 years of age or older and to live in ordinary housing. However, it turned out that FPs did not always know which of the patients registered with them had home care provided by a DN. This made it difficult for them to identify the last patient with home care by a DN in whose care they had been involved. They chose a patient that they thought was the last patient. Thus, for interviews 4–12 we instead requested that the patient should be a memorable patient, 65 years of age or older, in whose care they had been involved. The following analyses showed that the FPs were much more involved and updated concerning the care of these patients. For the second sample, a postal invitation was sent to 24 FPs in one city, and eight agreed to participate. During the analysis it became clear that the patient's age was of no importance, and it was therefore omitted as a selection criterion in interviews 6–13. In the first and second samples all but one FP worked in the same organisation as the DN. We then decided to invite FPs from a city where the FP and the DN typically worked in different organisations. Thus, for the third sample two candidates from that city were selected (at the suggestion of an FP who was familiar with that city), both of whom agreed to participate. In all, 13 interviews were performed. To obtain variation, the FP in interview 13 was again asked to talk about the last patient in whose medical treatment he had been involved who had home care provided by a DN. After 13 interviews saturation was judged to be reached and no more essential information was discovered. In GTM, however, this is typically a judgement with a certain amount of subjectivity.

### Data collection

Face-to-face semi-structured interviews, lasting 45–90 minutes, were performed by the author (SM) who issued the invitations. They were carried out in the FP's office at the healthcare centre. At the start of the interview the interviewer asked for informed consent. The FP was asked to chose a patient in accordance with the invitation letter. Based on an interview guide the FPs were asked to give a description of the patient, of the last time they were involved in the care, how long they had known the patient, what problems the patient had and how they were handled, what the patient could manage on his/her own, what the FP handled, who else participated in the care and what they handled, and how they cooperated with the FP. Finally, they were asked how they interpreted their role in the treatment of patients with home care provided by a DN and if they would like anything to be different.

During the interview, many of the FPs consulted the patient's medical and nursing records. Two of the FPs chose to talk about two patients in order to exemplify conditions they found problematic. Thus 15 patients were included.

Memos were written directly after each interview in order to capture the things that were not mentioned during the interviews such as if the FP appeared to be stressed or was late for the interview. The interviews were audio-taped and then transcribed verbatim. Before the next interview, the transcription of the previous interview was analysed to idenify important issues and questions that had arisen. New memos were written to document these issues as well as ideas about links between codes. These issues and questions were used to develop the follow-up questions in the initial interview guide in order to obtain greater understanding of the emerging process during the following interviews. The memos were not coded but were used as the basis for modifying the interview guide and as a basis for the questions posed to the data during the analysis.

### Data analyses

GTM was chosen because it is a method for studying social processes in areas where little is known [9-12]. Based on the GTM method, open, axial (theoretical) and selective coding was performed to enable the emerging theoretical model to be grounded in data. In the coding process the transcribed interviews were read and coded line by line to identify the different factors described by the FPs in the process of providing medical treatment for patients receiving home care from DNs, including how they had seen and experienced the process and how they had acted. Codes were generated to define different factors in the process, formulated in words used by the FPs. Through constant comparison of the codes, similar codes were detected and labelled. The data were read repeatedly to find variation in the codes that had been found, and to ensure that the codes were grounded in data. Codes with the same content and meaning were then grouped in descriptive categories. In this comparison of the codes we began generating the theoretical properties of the categories. The categories were sorted into higher-order categories and subcategories. The higher-order categories were subsequently compared with one another and concepts were formed, such as the concept of patients with reduced functional ability. Constant comparisons were carried out until saturation was judged to be attained. Through axial coding the concepts were related to each other. Patterns were analysed and a core process emerged through selective coding, linking all the concepts (the problems, the strategies used and the objective) to the core category, i.e. the effort to stay in charge.

All the authors were part of the research team that participated in the analysis process. The team comprised persons with different professional and research backgrounds (two FPs, a DN, a psychologist, a researcher specialised in GTM) in order to bring a wide variety of knowledge and preconceptions to the analysing process. Open coding was mainly done by the first author (SM), who is an FP, while the team members followed the progress and expressed their views. During the axial and selective coding, the other researchers participated actively in the coding process. The computer program NVivo was used during the process. In the presentation of the results, quotes are used to illustrate our findings. There is a number and a letter at the end of each quote. The number is related to factors about the patients, presented in table 2, and the letter is related to factors about the FPs, presented in table 1. The resulting model was discussed in a focus group with seven new FPs to validate the fit, relevance and work [11]. The project was approved by the Research Ethics Committee South, Karolinska Institute, Stockholm.

## Results

The core category developed in this study was the effort to stay in charge of the medical treatment, which involved three types of problems: gaining sufficient insight, making adequate decisions, and maintaining appropriate medical treatment. Three different categories of patients were distinguished where the FPs had difficulty staying in charge: patients with reduced functional ability, patients who were fixed in their ways, and patients with complex conditions. To overcome the problems, the FPs used four different strategies: relying on information from the DN and others, supporting close observation and follow-up by the DN and others, being constantly ready to change the goal of the treatment, and relying on the DN and others to provide treatment.

The views on and experience of providing medical treatment for patients receiving home care from DNs differed considerably in the different interviews.

"I think this is a relatively large part of what we do and an important part. I like it" 15 B – The interviewer: "How does it work, how can you manage his treatment?" "...to be honest, this is not really an ideal situation when the patient depends on your coming to their home. And there are so many actors involved in this. So, I mean, it's a problem, of course it is. It's not good. It's very hard to stay in charge" 9 D

### Three categories of patients where the FPs experienced problems in their effort to stay in charge

#### Patients with reduced functional ability

These patients were weak and had decreased initiative because of their medical condition, old age or reduced cognitive ability, or they had specific communication problems due to aphasia, language problems or dementia. Other types of reduced functional ability could include impaired vision and physical weakness. The result could be poorer ability to communicate, being unable to contact healthcare professionals when their health deteriorated, being unable to relate what had happened or how they felt. They could also be less able to manage their treatment, i.e. handle their medication and evaluate their condition and the effect of the treatment.

#### Patients who were fixed in their ways

These patients wanted to manage on their own, even when they needed medical attention according to others like the FP, the DN, home help personnel, family and friends. They did not contact healthcare professionals, they did not relate when they had problems, or they did not comply with recommendations from the FP, the DN or other care providers. Patients who were fixed in their ways became a problem when they had a medical condition and refused, for instance, to comply with recommendations concerning medical treatment. Examples of this behaviour included overusing or discontinuing prescribed medication, not wanting to adapt their home to home care, and refusing care outside the home.

#### Patients with complex conditions

These patients had complex medical conditions or a combination of medical and other problems that were difficult to handle. These problems included difficulty during investigation of their condition, such as when investigations could not be performed, did not give any answers, or when the information was ambiguous or hard to interpret. Also included here were difficulties with treatment or in getting medical advice. Patients in this category could also be those with alcohol abuse or overuse of painkillers, those with side effects from the appropriate treatment, or those approaching the end of life.

### Problems experienced by the FP in the effort to stay in charge

#### Problems in gaining sufficient insight

Gaining sufficient insight, such as when a patient needed medical attention because of a new disorder or deteriorating medical condition, was a problem when patients with reduced ability to communicate could not call for help or relate what was happening, or when patients who were fixed in their ways did not want to call for help.

Regarding a patient with dementia: The interviewer: "Does she contact you if she gets sick?" "...she used to do that herself but now she relies on her friends, they check on her"...6 B, "...Prednisolon was tested and, well, I felt that since her memory is bad it was very hard to say if she got better or worse..."3 B.

Regarding patients fixed in their ways: "I wanted that ...that they (the district nurses) would check on him...but he didn't want that, he wanted to manage on his own... he's very stubborn..."15 B

#### Problems making adequate decisions

Making adequate decisions was a problem when the patients had complex conditions or were fixed in their ways. When a condition was complex, the basis for making decisions was sometimes uncertain, such as when an investigation was difficult or gave no answers. Some FPs expressed concern that they had not done enough when the information was difficult to interpret.

The interviewer: "Is he still in much pain?" "...Well I think so, but it's hard to say..."5 A.

Decisions concerning adequate treatment could also be problematic in complex conditions like when patients were getting older and approaching the end of life.

"How closely should you follow up on an 80-year-old diabetic patient? ...You have to learn this on your own I suppose...It's much easier to see long-term factors when you have a young patient in front of you..."5 A.

When social welfare agencies failed in their attempts to treat alcohol abuse and judged they could do no more, and nursing homes would not accept a patient with abuse, then home care was described as the last resort. Under such circumstances, decisions concerning home care provided by DNs were problematic.

"At one time they didn't want to go there...sometimes he had friends there who abused alcohol, and once there were a lot of swords on the table, which they found a bit scary..."2 C.

Decisions concerning adequate treatment/medication when a person was regularly intoxicated were also difficult.

"...during one period he was fast asleep when they were supposed to give him his medication so they couldn't give it to him..." The interviewer: "Because he was drunk or...?" "Yes, we've decided that we can't give him any medication then, it's not possible" 2 C.

Making adequate decisions when the patients were fixed in their ways was also challenging, such as providing medical treatment and care when the patient refused changes in the home that were considered necessary in order to provide home care.

"He was bedridden...they lived in a flat that was full of trinkets...they had a double bed that was this low and then we couldn't solve the nursing situation" 10 A.

When attempts to influence the patient failed, the FP felt uncertain of what to do.

"Cutting down (on the painkillers) isn't going to work...She gets abstinence...every time I try...all hell breaks loose...So I thought I should really send her to the geriatric clinic for inpatient care in order to cut down, but she doesn't want that. I've tried to talk to her...what should I do? This is abuse really, or overuse." 1 B.

#### Problems maintaining appropriate medical treatment

Maintaining appropriate treatment for patients with reduced functional ability who could not handle their own treatment was a problem. For instance, when patients with reduced cognitive ability had other conditions such as infections, asthma or diabetes, or needed complex treatment, or when patients with reduced functional ability and impaired vision could not manage their medication.

"An 85-year-old woman with asthma, diabetes ...developing dementia...she's getting worse and worse...and even though the district nurse makes home visits every day, we're not sure that she's taking all her medications as she should or that she uses her inhalers the way she should..."6 B.

### Strategies used by the FPs in an effort to stay in charge of the medical treatment

In order to stay in charge of the medical treatment of patients with home care provided by DNs, the FPs had to gain sufficient insight, be able to make adequate decisions, and maintain appropriate treatment. It was found that four different strategies were used to overcome the difficulties: relying on information from the DN and others, supporting close observation and follow-up by the DN and others, always being ready to change the goal of the treatment, and relying on treatment provided by the DN and others.

#### Relying on information from the DN and others (Figure 1)

**Figure 1:**
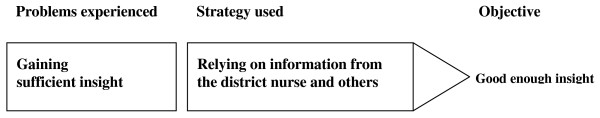
**Relying on information from others as a strategy for gaining sufficient insight**.

When the FPs had problems in gaining sufficient insight, they relied on the DN and the other care providers to alert them when necessary and to give them information.

The interviewer: "Who contacts you if she feels worse?"..."The district nurse, or her children, or home help personnel. Usually home help actually" 3 B.

They particularly relied on information from the DN, who in turn did examinations and tests in the home, supplied information about developments in the patient's medical condition, and information from other care providers, family and neighbours.

Some FPs expressed frustration. They missed the direct contact with the patient and found it difficult to stay in charge when they had to rely on information from others. Since the patients did not contact the FP themselves, information about the patient's own wishes and reactions was mediated through the DN and was consequently second- or third-hand information. Although this was the strategy that was used, some of the FPs felt uneasy about it as they did not always get the information they considered important.

The interviewer: "How do you feel looking after her works out?" "It's a bit uncertain, no grip on it. It's not like with people you have contact with yourself. It's like this with many patients with home care by district nurses, when it goes through another person, the district nurse and home help. You're not updated on how things are going, you have to trust your district nurse and hope they alert you at the right time..."4 B

The FPs did not always get information when there was a problem, and did not think they always needed to have that insight. They relied on the DN to alert them when necessary and to handle the situation adequately.

An old woman had fallen and had several fractures as a result. The district nurse found her and sent her to the emergency department 11 C.

The FP had sometimes wanted to have more insight or had felt that the DN had handled the condition in an improper way.

"She doesn't eat properly...forgets to eat...so the nurse provided nutritional beverages and contacted the home help personnel...eating doesn't work because they don't get any food when they can't manage to eat on their own, nobody makes sure the food gets to the mouth...then I feel that a nutritional beverage is the wrong solution..."8 B.

#### Supporting close observation and follow-up by the DN and others (Figure 2)

**Figure 2:**
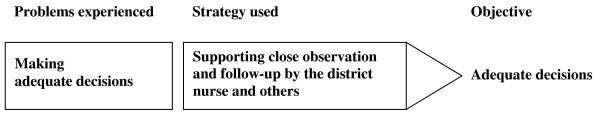
**Supporting close observation as a strategy for making adequate decisions**.

In complex circumstances where the FPs had problems making adequate decisions, they had to change their own role. In difficult care situations they supported the home care providers. In situations where the basis for decisions was uncertain or decisions concerning adequate treatment were problematic, they supported follow-up and close observation by others. Thus their own role was changed. The DN followed the patient's condition closely and assessed the risks. In such situations this was considered essential.

"...he is a heavy alcohol abuser...a lot of medications ...the district nurse provides a dose dispenser and home help gives him the medication... puts the medication there...then...checks if he's taken it. At one time he rarely did, and ended up in hospital again..." The interviewer: "What is your role?" "Well you can say that I'm really some sort of discussion partner and support for the district nurse, and then I try to do more, but it's difficult..."2 C.

Support of and follow-up by the family and the home help personnel was also essential.

"...We still feel that he needs nursing home care...we can't do anything as long as he doesn't want it...We try to support his wife...as soon as there's a problem I talk to the wife...I must say that we've given up a bit. It sounds terrible to say that, but that's how it is. We can't do more for him at the moment but it's important to support his wife, and then we'll see" 9 D.

An old woman had suffered from severe depression for many years. Only Lithium treatment had helped. Now she also had dementia and aphasia. Because of side effects from Lithium, a change in medication was discussed with several specialists who were uncertain about what to advise. The FP discontinued the Lithium and the woman was observed for both signs of depression and development of side effects by the home help personnel, who reported to the district nurse, who reported to the FP 4 B.

#### Being constantly ready to change the goal of the treatment (Figure 3)

**Figure 3:**
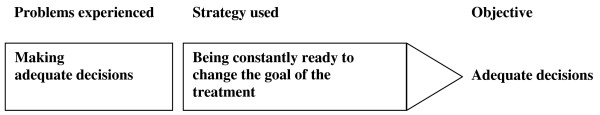
**Being constantly ready to change the goal of treatment as a strategy for making adequate decisions**.

In complex conditions when the basis for decisions was uncertain, the FP had to be ready to change and adjust the goal in relation to what was possible, and then rely on follow-up. Information from the follow-up constituted the basis for new decisions concerning the aim of the interventions.

"She...has some...kind of disease...a blood count that is sometimes as low as 80, that we don't understand in spite of massive investigation...Now we've reached a stage where we have no more therapeutic measures for her general illness, so to speak...we follow her blood count regularly...I've decided we won't get any farther with more investigations...our goal is for her to feel as good as she does and to maintain that" 12 C.

In complex conditions where deciding what constituted adequate treatment and how that treatment should be maintained was problematic, the FP had to evaluate the situation and be ready to adapt the goal to what was acceptable to the patient, possible to carry out, and medically appropriate. The FP had to rely on information from the follow-up by the DN and other home care providers in order to evaluate whether the plan was correct or had to be changed. The goal was to maintain the best possible state of well-being for the patient and to avoid risks.

The interviewer: "How long has he been your patient?" "...Since last January...he had more medication then...especially painkillers. He had an enormous amount, it was insane...it wasn't better, it was better to discontinue them, and his mind is much clearer and his intestinal tract functions..." The interviewer: "You and the district nurse have planned..." "Yes, together with his wife...we've tried to see if it works and it often does...I'd given him far too much and he wasn't better and he was affected by it, he lost his initiative..."9 D.

For patients approaching the end of life, the FP had to continuously evaluate the goal of the treatment, which was sometimes considered difficult. When the patient was treated at home, the FP was the only physician available.

"In the end you have to assume greater responsibility...since this is where the patient is...deciding if you should continue with more tests or....which I find relatively difficult...saying that this is enough and go on in a new direction..." The interviewer: "Stop the tests and start more palliative care?" "Yes, exactly..."10 A.

However, changing the aim of the treatment towards the end of life sometimes seemed natural to the FP.

"She was tired of life, she was ready and wanted to die...it was hard to argue...I asked her several times if she wanted...to move, but her answer was absolutely no. She hadn't left her apartment for five or six years and that was how she wanted it to be...she wanted to die at home, she was firm about that...and then she died...She had around-the-clock home help and the problems experienced by the family were solved...she had it her way...It was almost a story with a happy ending ..."14 D.

In difficult situations such as with patients with alcohol abuse, the FP sometimes did not know what to do. The FP and the DN had to evaluate the situation and the treatment and change the goal of the treatment in order to maintain the best possible state of well-being for the patient and avoid risks. When the patient objected to changes in the home to attain acceptable conditions for care, the FP had to assess what was acceptable to the patient, the FP and the care providers, and then adjust the goal of the treatment accordingly. This could mean referring the patient to other forms of care.

"... he had to go to the hospital...they both (the patient and his wife) agreed to this "10 A.

#### Relying on the DN and others to provide treatment (Figure 4)

**Figure 4:**
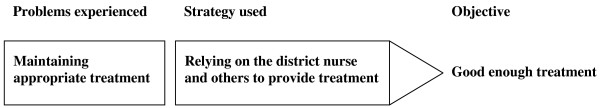
**Relying on others for treatment as a strategy for maintaining appropriate treatment**.

When the FPs had problems maintaining appropriate medical treatment they had to rely on the DN to provide the treatment as well as to assess and closely observe the patient's medical condition. Sometimes family members and home help personnel also assisted the patient with medication. The overall descriptions of the work of the DN and collaboration between the FP and the DN were very positive, and the FPs were confident about relying on the DN.

"I know that they are very competent...So I can rely on them" 8 B – "...the district nurse and the doctor have to be a team...that's a fundamental condition in order to call it home nursing..."3 B – "Without the nurse I would have had problems managing this, I think she's invaluable"13 C.

There were, however, exceptions to this rule, and some FPs expressed problems.

"We go through those patients who are relevant...I want to handle prescriptions in that context, not only get a list of prescriptions to be renewed...I want to know how the patient uses them, are they effective? That's almost impossible." "No, I don't know" 5 A.

### A comprehensive model of the effort to stay in charge (Figure 5)

**Figure 5:**
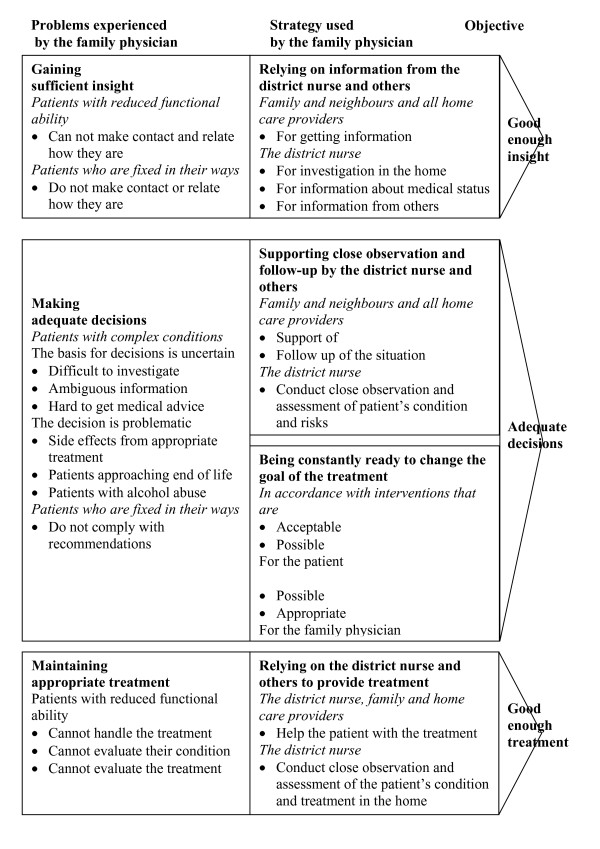
**Comprehensive model of the family physicians' effort to stay in charge of the medical treatment**. Comprehensive model of the family physicians' effort to stay in charge of the medical treatment of patients with home care provided by district nurses.

All the different strategies used for gaining insight, making adequate decisions, and maintaining appropriate treatment are presented in more detail in the comprehensive model of the effort to stay in charge.

When the model was presented to a focus group of seven new FPs, they said they recognised what was presented in the model; this was not new to them. What was new was that it was in writing, which had not been done previously. They stressed the importance of making the model understandable for persons who are not familiar with home care, so that the situation could be understood by others. They stressed that this was what made having ultimate responsibility for the medical treatment of these patients at home problematic, i.e. staying in charge of the medical treatment was problematic.

"I think this is part of our dilemma regarding home care provided by DNs. We are responsible for this ward in the home, but we have to rely on information from others. A lot of things happen that we don't see because it's not a ward where we can go and look at what's happening. Yet the ultimate medical responsibility is ours. That's probably why...not being in control of what happens to these patients is a problem for many conscientious doctors" (Focus group).

## Discussion

### Summary of findings

The FPs had ultimate responsibility for the medical treatment of patients with home nursing provided by the DN. When they talked about these patients and their medical treatment, the effort to stay in charge of the medical treatment emerged as a main concern. In order to stay in charge, the FP had to gain sufficient insight, make adequate decisions about interventions, and maintain appropriate treatment. This was a problem for the FP with regard to patients with reduced functional ability, patients who were fixed in their ways and did not ask for help or objected to recommendations by the FP, and patients with complex conditions. Four different strategies were used in the effort to stay in charge. These were: relying on information from the DN and others, supporting close observation and follow-up carried out by the DN and other care providers, being constantly ready to change the goal of the treatment, and relying on the DN and others to provide treatment.

### Having ultimate responsibility for medical treatment at home when staying in charge of the medical treatment is problematic

Gaining sufficient insight, making adequate decisions, and maintaining appropriate treatment are normal procedures involved in staying in charge of medical treatment when the FP has ultimate responsibility for the treatment. However, this process usually takes place in close cooperation with the patient during a series of consultations; Family Medicine involves "a unique consultation process, which establishes a relationship over time, through effective communication between doctor and patient" [13]. The problematic patients in this study had complex problems, and knowing how to handle them was difficult. In addition, the patients' ability and willingness to provide information, as well as their ability to manage their treatment and their willingness to comply with recommendations, were often decreased. The effort to stay in charge of the medical treatment, and the fact that they were responsible for the medical treatment in this situation, left the FPs with a feeling of uncertainty.

Another study revealed that the FP "found older people's multiple pathology complex and sometimes threatening" [8]. We found that this complexity included problems in making adequate decisions concerning the treatment of the condition itself, as well as in communicating with the patient and negotiating with the patient concerning how to solve a problem in a way that ensured that the patient received adequate treatment. In much of this the FP had to rely on others.

### Relying on others

The reduced functional ability of patients in home care is well known [3-5] and is one of the reasons they receive such care from the DN. What we identify in this study is the way in which this has implications for the FP's ability to stay in charge of the medical treatment. During consultations with patients at the healthcare centre, the FP and the patient communicate and the FP assesses the condition and the situation. For the patients considered in this study, the FP often had to rely primarily on second- or third-hand information from the DN and other care providers, and on help with treatment, observation and assessment, in order to gain sufficient insight and to maintain appropriate medical treatment. We have not found any previous studies concerning this issue. Most studies focus on different ways of determining the patient's health status, functional ability or need of care [14-16].

Different aspects of family medicine – coordinating care, working with other professionals in the primary care setting, and managing the interface with other specialities – [13] are clearly seen in the care of these patients, especially the close collaboration between the FP and the DN. This collaboration is not always uncomplicated, as different professions may have different views and strategies for handling a problem [17,18]. Some difficulties emerged in this regard, but most of the FPs in the present study expressed feeling satisfied with their collaboration with the DNs.

Freedom of choice and respect for autonomy are essential factors in Scandinavian society and are considered the foundation for how care and treatment should be carried out [1]. However, problems were experienced in trying to stay in charge of the medical treatment of patients who did not ask for help and who wanted to manage on their own, even when their health deteriorated. The FPs had to rely on the persons who saw the patient on a regular basis and could notice when the patient was in need of a change in medical treatment, and could try to persuade the patient to accept an intervention by the FP.

### Readiness to continuously change the goal of the treatment

Evaluating and diagnosing a medical condition and its seriousness and making a decision about treatment is part of the daily work of the FP [19-22]. The process of diagnosing is complex in itself [23]. It is even more complex for patients with several different medical and functional problems, where what is acceptable, possible and appropriate under the circumstances has to be taken into account. Thus the FP has to be ready to change the goal of the treatment and adjust it to what the patient can accept, and what is medically appropriate under the circumstances. Such difficulties have been described in similar situations. A study of the problems that the FP encounters in palliative care showed that they constitute complex challenges [5]. The complexity of caring for dying patients has been illustrated by a theoretical three-dimensional model developed in order to help FPs define their role [24].

As described above, the FP's basis for evaluation and diagnosis was dependent to a large extent on information from the DN and other home care providers. They provided the FP with the information, based on their assessment of the situation, that they considered relevant and important for the FP to be aware of. Thus, in their evaluation the FPs in our study had to take into account both medical and non medical factors, which to a large extent constituted second- or third-hand information. They talked about a feeling of uncertainty, and about how they handled this by continuously evaluating the goal of the treatment as new information emerged about the situation.

### Supporting close observation and follow-up by the other care providers

Supporting the family, as well as other care providers, is part of the FP's role [24-27]. In our study the FPs talked about how they supported close observation and follow-up care provided by the DN and others, not only because they needed this support but also because this was sometimes the only way the FPs could stay in charge of the medical treatment.

### Strengths and weaknesses

The strength of our study is that we have been able to identify and describe a complex process and create a model that makes the process easier to understand. GTM is a method well suited for studying social processes and for creating models grounded in data [9,10,12]. Thus the model is relevant for the situations and the patients that these FPs talked about. As is always the case with GTM, further research is needed in order to see if the model is relevant in other situations and for other patients with home care provided by the DN. The focus group recognised the results of the study, both the problems involved in staying in charge of the medical treatment and the process used by the FPs in their effort to do so. What is of importance is that for the first time the problems and the process were identified and put in writing.

It is both a weakness and a strength that the interviewer (SM) is a FP and that she interviewed her own peers. A majority of those who agreed to participate had had previous contact with the interviewer. Earlier studies have found that it is easier to gain access to FPs in order to interview them if the FP interviewer is known to the respondents. However, the relationship between the interviewer and the respondent can influence the data. If the interviewer is seen as a peer and confidant, this results in richer and more personal accounts of attitudes and behaviour. But it can also influence the researcher's ability to obtain data, as there might be a shared understanding that the interviewer does not explore [28,29].

Factors that influenced the theoretical sampling concerned both the FPs and their working conditions and the patients. Initially, a subpopulation of FPs working in larger cities were invited to participate and were asked to talk about the last patient in whose care and treatment they had been involved who met the criteria. As data were analysed, priority was given to including FPs with different working conditions that might have influenced the FP's way of working as it appeared in the interviews, and the FPs were asked to talk about a memorable patient. The patients who were then included had complex and memorable problems in which the FP was deeply involved. This provided richer data than if a less memorable patient receiving home care from the DN had been chosen. However, problems arising from less active involvement by the FP that were detected in some of the earlier interviews would have been missed.

## Conclusion

The patients in this study differed from most patients seen at the healthcare centre as the consultation with the patient could not provide the usual foundation for decisions concerning medical treatment. Information from and collaboration with the DN, in particular, but also other home care providers, was essential for the FP's effort to stay in charge of the medical treatment. The complexity of the situation made it problematic for the FP to make adequate decisions about the goal of the medical treatment. The goal of the treatment had to be constantly evaluated based on information from the DN and other care providers, and thus this information was absolutely crucial.

### Practical implications

How FPs manage the medical treatment of patients with home care provided by DNs has to a large extent been an unknown and often critisised process, at least in Sweden. Identifying problems encountered by the FPs and their strategies for handling them contributes to the knowledge about this process. This enhanced knowledge has practical implications when development and organisation of the care for patients with home care by DNs is discussed both locally and on a nationel level. It can also help the individual FP and DN to understand the problems they encounter in the everyday care and treatment of these patients.

The strategies depicted in this study might also be applicable in other situations where indirectly managed care is being provided.

### Implications for future research

These results point to the need for more in-depth study of the process involved in the collaboration in home care between the FP and other care providers, especially the DN. This process is of fundamental importance regarding the possibility for FPs to provide medical treatment of good quality for these patients. The results also point to the need to study what happens when there are problems in using different strategies and when the objectives of these strategies are not met. This study explores the situation from the FP's point of view. As there are many actors involved in this process, it would be of interest to explore it from the point of view of the patient, the DN and the other care providers who are involved.

## Competing interests

The authors declare that they have no competing interests.

## Authors' contributions

SM and AKF were responsible for the original idea. SM designed the study with some external help, issued all the invitations and conducted all the interviews, and was responsible for the initial analyses. SM wrote the first draft and subsequent revisions of the manuscript. All authors have been involved in the data interpretation, in critically revising the manuscript in terms of important intellectual content, and all of them have read and approved the final version of the manuscript.

## Pre-publication history

The pre-publication history for this paper can be accessed here:


